# Effectiveness of Flattening-Filter-Free versus Flattened Beams in V79 and Glioblastoma Patient-Derived Stem-like Cells

**DOI:** 10.3390/ijms24021107

**Published:** 2023-01-06

**Authors:** Valentina Dini, Giuseppe Esposito, Andrea Sacconi, Marco D’Andrea, Maria Antonella Tabocchini, Pasquale Anello, Lucia Ricci-Vitiani, Mariachiara Buccarelli, Roberto Pallini, Lidia Strigari

**Affiliations:** 1 National Center for Innovative Technologies in Public Health, Istituto Superiore di Sanità, 00161 Rome, Italy; 2 Istituto Nazionale di Fisica Nucleare (INFN), Sezione di Roma 1, 00185 Rome, Italy; 3 UOSD Clinical Trial Center, Biostatistics and Bioinformatics, IRCCS Regina Elena National Cancer Institute, 00144 Rome, Italy; 4 Laboratory of Medical Physics, IRCCS Regina Elena National Cancer Institute, 00144 Rome, Italy; 5 Department of Oncology and Molecular Medicine, Istituto Superiore di Sanità, 00161 Rome, Italy; 6 Institute of Neurosurgery, Università Cattolica del Sacro Cuore, IRCCS Fondazione Policlinico A. Gemelli, 00168 Rome, Italy; 7 Department of Medical Physics, IRCCS Azienda Ospedaliero-Universitaria di Bologna, 40138 Bologna, Italy

**Keywords:** glioblastoma-like cell lines, V79 cells, DNA damage, dose rates, flattening filters

## Abstract

Literature data on the administration of conventional high-dose beams with (FF) or without flattening filters (FFF) show conflicting results on biological effects at the cellular level. To contribute to this field, we irradiated V79 Chinese hamster lung fibroblasts and two patient-derived glioblastoma stem-like cell lines (GSCs—named #1 and #83) using a clinical 10 MV accelerator with FF (at 4 Gy/min) and FFF (at two dose rates 4 and 24 Gy/min). Cell killing and DNA damage induction, determined using the γ-H2AX assay, and gene expression were studied. No significant differences in the early survival of V79 cells were observed as a function of dose rates and FF or FFF beams, while a trend of reduction in late survival was observed at the highest dose rate with the FFF beam. GSCs showed similar survival levels as a function of dose rates, both delivered in the FFF regimen. The amount of DNA damage measured for both dose rates after 2 h was much higher in line #1 than in line #83, with statistically significant differences between the two dose rates only in line #83. The gene expression analysis of the two GSC lines indicates gene signatures mimicking the prognosis of glioblastoma (GBM) patients derived from a public database. Overall, the results support the current use of FFF and highlight the possibility of identifying patients with candidate gene signatures that could benefit from irradiation with FFF beams at a high dose rate.

## 1. Introduction

Patients with newly diagnosed glioblastoma (GBM) are treated with maximal safe resection followed by radiotherapy (RT) plus concomitant and maintenance chemotherapy. The chemotherapy regime includes temozolomide (TMZ) as the first-line treatment and either bevacizumab or nitrosoureas, which serve as second-line therapy [1,2]. Despite these treatments, the overall survival (OS) of GBM patients is commonly short and without effective therapy, rarely exceeds a few months.

Tumor-Treating Fields (TTFields) are emerging radiation treatment modalities for GBM, which has shown a good safety profile and better efficacy in newly diagnosed GBM, while a less clear effect was shown for recurrent GBM [3].

In recent years, interest in FLASH radiotherapy has been growing, mainly due to the radiobiological advantage of instantaneous dose rates observed in healthy tissue, although it is not reported on tumors. Preclinical studies indicate that ultrahigh dose rate FLASH radiotherapy delivery of radiation might reduce normal tissue toxicity without compromising GBM tumor response [4,5].

Currently, the high dose rate, available from modern accelerators, is the best-performing modality adopted in clinical RT practice. The wide diffusion of irradiation techniques, such as intensity-modulated radiotherapy (IMRT) and volumetric-modulated arc therapy (VMAT), particularly when flattening-filter-free (FFF) beams are adopted, demands further radiobiological investigations due to their non-trivial time structure.

In IMRT, delivery time increases compared to 3D conventional treatments, thus potentially reducing the therapeutic effect of radiation [6,7]. VMAT treatments reduce the treatment time and potentially improve tumor control. FFF beams allow up to a four-fold increase in the dose rate, thus reducing the probability of the patient’s motion and increasing his comfort. At state of the art, FFF is a technique already experienced in clinical settings in Stereotactic Body Radiation Therapy (SBRT) of vertebral, lung, liver, or intracranial tumors [3] thanks to the generated high gradient dose distribution hence the benefit of limiting normal tissue damage while preserving the tumor control rate.

At the same time, literature data on the potential biological effects of high-dose-rate delivery of FFF beams showed conflicting results on biological effects at the cellular level [8,9,10,11,12,13,14,15,16,17]. Indeed, some studies did not show any significant difference in cell survival in different populations (healthy or cancer cell lines) irradiated at conventional or high dose rates [9,10,11,12,13,14,15]. Other authors indicate a preference for RT delivered in high-dose-rate FFF regarding tumor-cell death and healthy tissue sparing [16,17]. One of the reasons for these contradictory findings is likely due to the dosimetric differences in experimental setups, which make it challenging to carry out conclusive results. Thus, we decided to conduct an additional methodological study.

We used conventional beams with both the flattening filter (FF) and FFF generated by the same 10 MV TrueBeam^TM^ (Varian) linear accelerator to irradiate V79 Chinese hamster lung fibroblasts. The aim was to investigate the possibility that irradiation with beams differing in dose rates (4 Gy/min and 24 Gy/min FFF) and PRF could result in a different radiobiological response as assessed by the clonogenic assay.

V79 cells represent a reference cell line widely used in radiobiological studies, including radiation beam characterization. The survival curve for V79 cells exposed to sparsely ionizing radiations has a broad initial shoulder that makes them very responsive to small changes in the radiometric characteristics of ionizing radiation beams.

In addition to early survival, we also studied late survival to assess the possible presence of genomic instability (lethal mutations) in the progeny of irradiated cells [18,19]. To our knowledge, this is the first time this endpoint has been studied in V79 cells after irradiation with FFF and FFF beams.

To strengthen the novelty of our study, we noted that the types of cells generally used for these studies are not derived from patients. Here, we used, for the first time, glioblastoma stem-like cells (GSCs) derived from patients and modern FFF beams to evaluate their radiobiological response in terms of cell killing and DNA damage. Two cell lines, named line #1 and line #83, derived from two patients with “poor” and “very poor” clinical outcomes, respectively, were irradiated with FFF beams at either 4 Gy/min or 24 Gy/min dose rate. GBM (World Health Organization grade IV glioma) is one of the most common, malignant, and lethal primary brain tumors associated with a very poor prognosis. The high recurrence rate and the failure of conventional treatments in GBM patients seem to be related to radioresistant stem cells inside the tumor mass. The high recurrence rate has been related to the activation of a DNA damage response, leading to a better repair capability [20,21].

Our findings support the use of RT with FFF beams also for GBM patients and suggest the biological features of these tumors (e.g., gene expression profiling as prognostic signature) to predict the patient outcome after irradiation with modern accelerators.

## 2. Results

### 2.1. Cell Killing and Lethal Mutations in Chinese Hamster V79 cells

**Figure 1:**
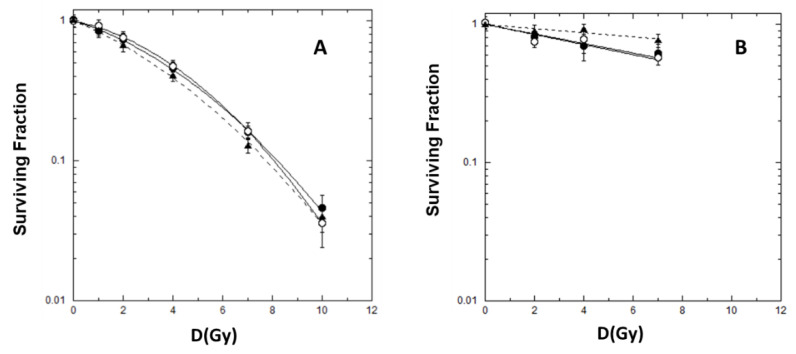
Dose–response curves for early and late survival of Chinese hamster V79 cells irradiated with 4 Gy/min-FFF (solid circle), 4 Gy/min-FF (open circle), 24 Gy/min-FFF (solid triangle): early survival (panel (**A**)), late survival (panel (**B**)). The error bar represents the standard error of the mean (SEM) coming from 3 independent experiments for each irradiation condition used.

Survival curves for early and delayed reproductive cell death, representative of cell killing and lethal mutation induction, respectively, are shown in Figure 1. The best-fit parameters, obtained using Equations (1) and (2), are reported in Table 1.

We obtained linear quadratic curves for early cell survival in the dose range 1–10 Gy. No significant differences were observed as a function of the dose rate (4 vs. 24 Gy/min) or the pulse repetition rates (FF or FFF).

For late survival, we obtained roughly linear relationships in all cases. At the dose rate of 4 Gy/min, the same alpha parameters were obtained irradiating with FF and FFF beams; a decrease in the α parameter value, although without reaching a significant difference, was observed after FFF beam irradiation at the dose rate of 24 Gy/min.

### 2.2. Cell Killing for GSCs

**Figure 2:**
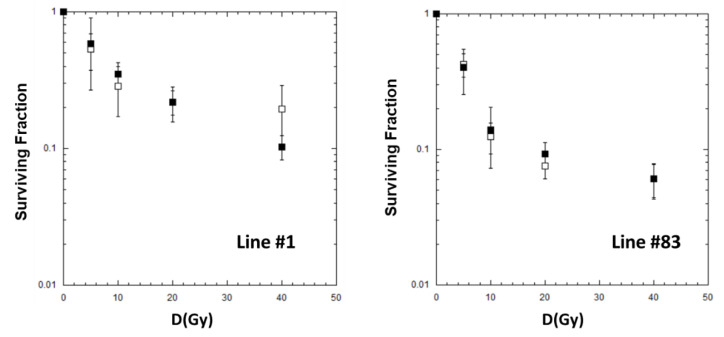
Dose–response curves for GSC cells irradiated in FFF conditions at different dose rates: 4 Gy/min (solid square), 24 Gy/min (open square). The error bar represents the standard error of the mean (SEM) coming from at least 2 independent experiments for each irradiation condition used.

GSC line #1 and line #83 were irradiated with FFF beams at 4 Gy/min e 24 Gy/min in the dose range 5–40 Gy and analyzed for survival as described in Materials and Methods. The survival curves of the two cell lines showed a biphasic dose–response relationship for both dose rates, with a steep initial slope that tends to a shallower curve at higher doses (Figure 2).

This trend, already observed in the literature [22,23,24], is usually related to the presence of two or more subpopulations of cells with different radiation sensitivity. Using Equation (4) (see Materials and Methods), we obtained the best-fit parameters reported in Table 2.

For both cell lines, #1 and #83, similar survival levels for each dose point were observed as a function of the dose rate. However, in the range 10–40 Gy, cells of line #83 seem to include a larger fraction of a more radiosensitive subpopulation than cells of line #1.

According to the model (Equation (4)), the radiosensitive subpopulation (f) in line #1 ranges from 56% to 80% of the entire cell population, while in line #83 the radiosensitive subpopulation ranges from 88 to 93% (Table 2). As for the α_s_ and α_r_ parameters, no significant differences are observed in both cell lines. Table 2 also shows large fluctuations in the errors of the fit, especially for line #1.

### 2.3. γ-H2AX Analysis by Flow-Cytometry

**Figure 3:**
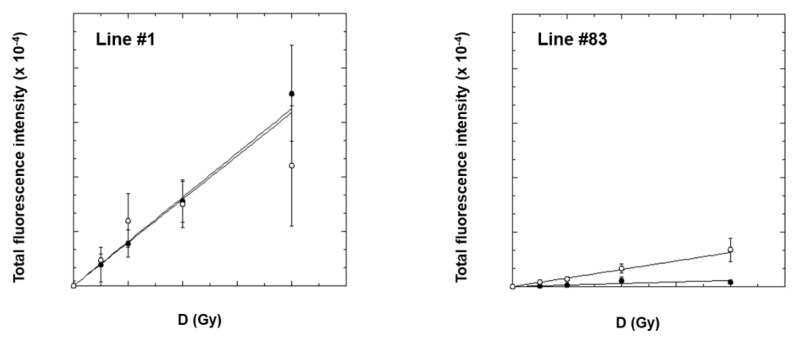
Dose–response curves for γ-H2AX induction (as measured as γ-H2AX immunofluorescence intensity) in GSC line #1 and line #83, 2 h after irradiation with FFF beams at different dose rates: 4 Gy/min (solid circle), 24 Gy/min (open circle). The error bar represents the standard error of the mean. Immunofluorescence intensity values in unirradiated cells (namely ~4.0 and ~2.1 a.u. for line #1 and line #83, respectively), have been subtracted from the values of irradiated samples.

The dose dependence of γ-H2AX induction using FFF beams at 4 Gy/min e 24 Gy/min in GSC line #1 and line #83 was evaluated after irradiation with different doses up to 40 Gy. We used the total γ-H2AX immunofluorescence intensity, determined at 2 h after irradiation (i.e., the time corresponding to the maximum fluorescence values for both lines), for evaluating the induced initial DNA damage. As shown in Figure 3, for both cell lines a linear dose dependence was observed at both dose rates. Much more radiation-induced damage was apparently present in line #1 compared to line #83.

The dose–response curves for line #1 show the same slope at the two dose rates, i.e., (2.40 ± 0.40) × 10^4^ Gy^−1^ For line #83, a big difference was found instead; the slope at 24 Gy/min was much higher than that at 4 Gy/min (i.e., (0.47 ± 0.06) × 10^4^ Gy^−1^ and (0.08 ± 0.02) × 10^4^ Gy^−1^, respectively).

We also investigated the de-phosphorylation of γ-H2AX in GSC line #1 and line #83 after the dose of 20 Gy (see Supplementary Material Figure S1) at 4 h and 24 h after irradiation by measuring the Persistence Ratio (PR, see Materials and Methods), representing the % of residual DNA damage, whose values are shown in Table 3 for both GSCs.

At 4 h, the PR values at both dose rates for the two GSCs show no significant differences, although the PR values for line #83 are slightly higher than those for line #1. On the other hand, the 24 h PR values increase with increasing dose rates for both GSCs. This indicates that fluorescence (i.e., DNA damage) persists longer by irradiating at 24 Gy/min than at 4 Gy/min.

### 2.4. Gene Expression Analysis and Potential Prognostic Factors for ‘Patients’ Survival

**Figure 4:**
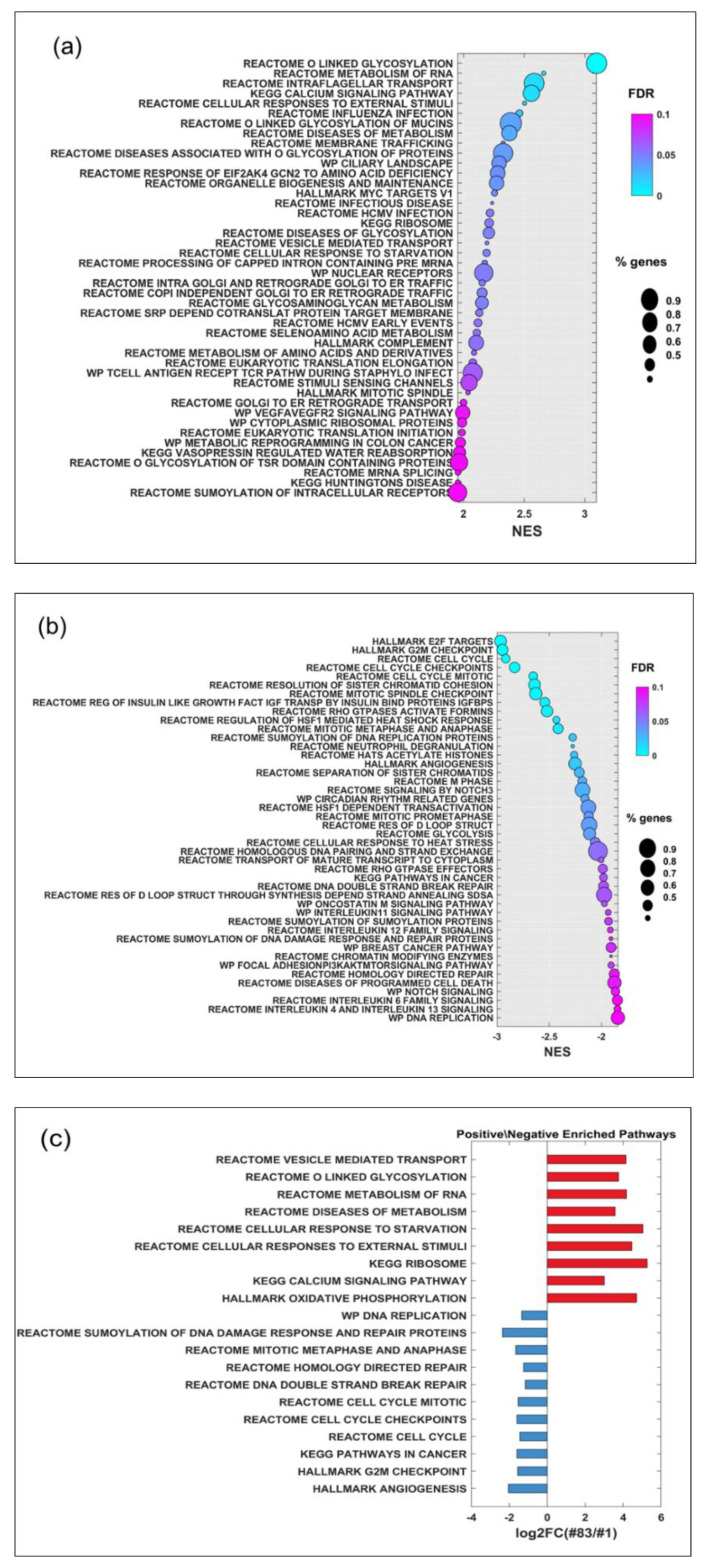
(**a**) Positive and (**b**) negative enrichment pathways of line #83 versus line #1 sorted using the FC of each gene. (**c**) A subset of positive in red and negative in blues enrichment pathways of line #83 versus line #1 potentially related to the radiation-induced effects. The average expression of the genes included in each pathway was evaluated to assess FC between the two lines.

Figure 4a,b shows the positive and negative enrichment pathways of line #83 versus line #1 by using sorted fold changes (FC) of each gene between the two lines, respectively.

A subset of these pathways potentially related to the radiation-induced effects is shown in Figure 4c. Most of these pathways are related to DNA repair, checkpoint, and cellular cycle regulation. The positive-enriched pathways include: reactome vesicle mediate transport, reactome or linked of glycosylation, reactome metabolism of RNA, reactome disease of metabolism, and hallmark oxidative phosphorylation, while the negative enriched pathways comprise: reactome DNA double-strand breaks (DSB) repair and reactome sumoylation of DNA damage response and repair protein.

**Figure 5:**
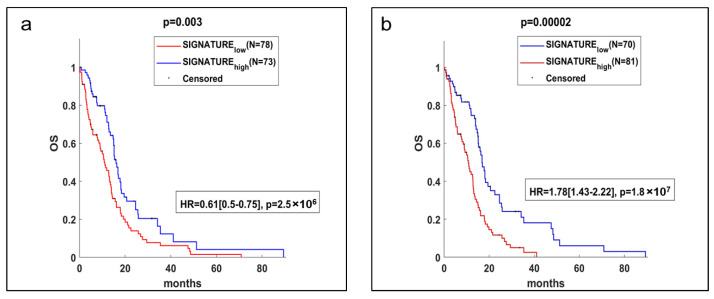
Prognostic gene signatures from DOWN (panel (**a**)) and UP (panel (**b**)) enriched pathways evaluated on TCGA GBM. The gene signatures were obtained considering all prognostic genes included in the positive- or negative-enriched pathways. Patients were split in low and high expression of the signature based on the negative or positive z-score of the mean genes expression, respectively. N = 151.

Figure 5 shows the Kaplan–Meier analysis of the overall survival of identified GBM virtual The Cancer Genome Atlas (TCGA)-based patient cohort, grouping the patients with gene signatures identified from up- and down-enriched pathways (listed in Figure 4c) in panels a and b, respectively. TCGA-based patient cohort with high and low gene signature shows a prognosis similar to one of the patients from which line #83 (red curve) and line #1 (blue curve) was derived, respectively.

The overall survival of GBM patients grouped according to the expression of the signature based on the negative z-score of the mean gene expression (including NCOA4, NBN, STAG2, MAPRE1, E2F5, PTEN, and CEP57) is statistically significantly different with a hazard rate of 0.61 (95% confidence interval: 0.5–0.75), as shown in Figure 5, panel a. The group with high expression of the signature based on the negative z-score of the mean gene expression (blue line) mimics the “poor” prognosis of the patient from which line #1 was derived.

Similarly, the difference in overall survival of GBM patients grouped according to the expression of the signature based on the positive z-score of the mean gene expression (including GALNT12, POR, RPL39L, CUL1, MAP2K3, CAMK4, DENND2A, HPS1, ADAMTSL1, SCO1, GPC5, GALNT6, and MYO1C) is statistically significantly different, with a hazard rate of 1.78 (95% confidence interval: 1.43–2.22), as shown in Figure 5, panel b. The group with high expression of the signature based on the positive z-score of the mean gene expression (red line) mimics the “very poor” prognosis of the patient from which line #83 was derived.

## 3. Discussion

After the introduction of intensity-modulated beams in clinical practice, several authors investigated the effect of fraction delivery time and dose rate mimicking IMRT treatments on cell survival. Fraction delivery time was found to be statistically correlated with reduced cell survival [6,25], and protracted delivery times of up to 15–20 min have been reported to increase in vitro cell survival by up to 20% due to sublethal damage repair [26,27,28]. In contrast, a reduced cell survival after an irradiation time of 1 min compared to the same dose delivered in 5/10 min has been found in [26,29,30], demonstrating a very fast cellular response to irradiation that is of radiotherapeutic significance.

The increase in the dose rate of FFF beams is expected to overcome the loss of radiation effectiveness due to beam modulation expected during IMRT treatment with beams at fixed angles [31,32]. FFF beams allow treatment delivery at a higher dose rate, counteracting the lengthened treatment time due to frequent beam interruptions during gated RT [13].

A literature survey witnessed considerable interest in the subject, and encouraging results have been obtained.

Bewes et al. [6] reported a significant trend to increase survival in melanoma cancer cells while decreasing the average dose rate and keeping the total dose constant. Bewes et al. [6] found increased cell survival at extended delivery times.

Sørensen et al. [9] compared the effects of three different dose rates (i.e., about 5, 10, and 30 Gy/min, with dose rates in the pulse of 56.5, 112.8, and 338 Gy/s) on clonogenic survival of V79 cells and FaDu (DD) tumor cell line and did not report any radiobiological difference in cell survival for doses up to 10 Gy. However, they obtained dose rates comparable to that of FFF beams, using a flattened beam from a conventional linac and reducing the target-to-cell distances. This arrangement ensured that any changes in cell response were purely the result of changes in the dose rate of the beam, while the effects of other differences (e.g., beam energy spectrum) between FFF and FF beams were not considered.

In contrast, Lohse et al. [11], also using FFF X-ray beams from a Varian TrueBeam Linac, reported reduced clonogenic cell survival for two commercial GBM lines, namely the T98G (p53 mut) and U87-MG (p53-wt), irradiated with increasing dose rates (between 0.2 Gy/min and 24 Gy/min). They observed a statistically significant difference in cell killing at the dose of 10 Gy. Of note, quantitative differences depended on both p53-status and dose rate.

Karan et al. [12] investigated the V79 cell line and two p53 wild-type human cell lines, namely H460 non-small lung carcinoma cells and SiHa cervical carcinoma cells. They found no significant difference between survival fractions for FF versus FFF beams after 5 or 10 Gy acute irradiation. An increase in survival was seen in both FF and FFF modes upon protracting dose delivery to 15, 30, or 60 min rather than delivering acutely. Furthermore, the analysis of induced DNA DSB via the γ-H2AX assay showed no difference between FF and FFF beams. Regarding the biological effect of the high dose rates associated with FFF beams, the authors modified, via a compensator, the fluence of the 6FFF output of a Truebeam linac in order to generate a homogenous dose profile across the cell monolayer, and this setup is expected to have changed the spectral composition and local dose rate of incident FFF beam.

Verbakel et al. [10] explored the radiobiological effects of FFF irradiation with a dose rate of 2400 MU/min and four times higher doses per pulse compared to irradiation with FF beams (600 MU/min). They found equal clonogenic cell survival for three human cancer cell lines, the astrocytoma cell line D384, the malignant glioma cell line T98, and the small cell lung cancer cell line SW1573, after single fraction irradiation up to 12 Gy, and for the D384 and SW1573 cell lines after fractionated irradiation as well. They used, however, an alternative approach, where a dynamic IMRT technique was applied to improve the agreement between the flattened and FFF beam dose profiles. As with previous studies, the authors delivered the photons from a static gantry angle and applied a sliding window IMRT plan with the flattened beam to generate a dose profile similar to a FFF beam. However, the use of the FFF beams with an IMRT approach produced a 20% decrease in the dose rate on the cell monolayer compared to a static filtered field.

Lasio et al. [14], using FFF 10 MV using settings similar to the one used in the present study, compared the effect of the higher dose rates achieved with FFF beams on the clonogenic survival of V79 cell line and two commercial GBM cell lines (T98G and U87-MG). The survival fraction for any of the cell lines considered was not statistically different as a function of dose-per-pulse, average dose rate, or total dose delivered.

Dubois et al. [33], with irradiation methodologies similar to the ones we used, assessed clonogenic survival in several tumors and normal cell lines and found no difference in cell survival when irradiation was performed with 10 MV FFF at the dose rate of 24 and 4 Gy/min and FF at 4 Gy/min, respectively, in the dose range 2–10 Gy.

Hao et al. [34] have assessed the therapeutic effect of 2, 4, and 6 Gy X-irradiation at dose rates of 4.2 Gy/min or 21.2 Gy/min on GSCs isolated from xenografts. The authors analyzed different endpoints (i.e., cell viability, tumor sphere formation, clonogenic survival, cell cycle apoptosis, and DNA damage). However, there was no difference in GSCs responses regardless of the dose rate.

Laurent et al. [35] have studied the effects in vitro of a high dose rate (4, 12, or 24 Gy/min using the FFF mode) on the anti-tumor immune response in CT26 murine colon cancer cells. The activation of the anti-tumor immune response was evaluated by the induction of genes of the type I interferon pathway by RT-qPCR and by the induction of immunogenic death biomarkers. The authors have not observed any significant difference in the induction of genes of the type I interferon pathway and the studied immunogenic death markers according to dose rate irradiation.

Sarojini et al. [17] reported that a dose rate of 2400 MU/min enhances apoptosis in WC00046, WC00060, and WC00081 melanoma cells through a Fas-mediated apoptotic pathway, suggesting a potential antimelanoma therapy by using a combination of high dose-rate (2400 MU/min) and low total dose (0.5 Gy). This enhances the radiosensitivity and apoptotic rates in melanoma cells while preserving the survival of primary human epidermal melanocytes (HEM) cells.

The heterogeneity of irradiation setups and methodologies are summarized in Table 4 and the different cell models used so far suggested further investigations.

In this context and to the best of our knowledge, our work represents an original article to improve the current knowledge on how the radiobiological response in V79 cells (used as a reference line) and non-commercial primary GSCs (i.e., derived from patients), can be influenced by high-dose FF and FFF irradiation.

V79 cells exposed to photons (X- or γ-rays) provided stable results over time and have been extremely useful to compare different beam types and irradiation modalities [36,37]. We used the clonogenic assay to investigate the possibility that irradiation at a Varian TrueBeam accelerator with beams having different dose rate beams (4 Gy/min and 24 Gy/min with FFF) and PRF (FFF vs. FF) could result in a different radiobiological response.

According to our results, the clonogenic survival of the V79 cell line exposed to a clinical beam dose rate of 4 and 24 Gy/min in the FF or FFF condition was not affected by either the absorbed dose or the deposition pattern. This result is in agreement with the data already mentioned by Sorensen et al. [9], Karan et al. [12], and Lasio et al. [14]. Moreover, in the same cell line, we also investigated the clonal progeny of irradiated cells in terms of delayed reproductive cell death, an endpoint related to genomic instability. This endpoint represents a non-targeted effect, possibly resulting in additional damage amplifying the biological effectiveness of a given radiation dose on normal tissues. Our results showed that surviving fraction in the progeny of directly irradiated cells decreases in a dose-dependent fashion, giving evidence of genomic instability. A decrease was registered in the surviving fraction as a function of absorbed dose at the dose rate of 4 vs. 24 Gy/min, a trend suggesting a radiobiological advantage.

Furthermore, we also exposed two GSCs to dose rates of 4 and 24 Gy/min FFF 10 MV photon beams using the same Varian TrueBeam accelerator. These lines (i.e., line #1 and line #83), derived from two patients with “poor” and “very poor” prognoses, respectively, have been previously well characterized in terms of gene, proteomic, and metabolic patterns (summarized in Supplementary Materials Table S1). In particular, gene expression data for the two lines were collected, and unsupervised hierarchical clustering of the samples using the 1000 most variable genes/transcripts. This analysis produced two distinct GSC clusters, reminiscent of the separation into GSf (full stem) and GSr (restricted stem) phenotypes previously reported by Günther et al. [38] and by Schulte et al. [39]. Line #83 falls into the phenotype GSr-like, characterized by a more mesenchymal-like phenotype, no CD133 expression, adherent growth in vitro, and low invasive behavior in vivo. Line #1 falls into phenotype GSf-like characterized by a pro-neural-like gene expression signature, growth as floating spheres in vitro, expression of CD133, and high invasiveness in vivo. These clusters closely overlapped those obtained both from metabolic analysis by NMR Spectroscopy [40].

Using the classification of Gobin et al. [41], we also found that the signatures of cell lines #1 and #83 can be included in the group G3 and G1, respectively, further confirming that these cells are representative of patients with “poor” and “very poor” prognosis (see Supplementary Materials Figure S2).

The survival curves for cell lines #1 and #83 show similar survival levels with a biphasic dose–response relationship for both dose rates, with a steep initial slope that bends to a shallower curve at higher doses (Figure 2). Moreover, line #1 appears to be more radioresistant than line #83 at doses higher than 10 Gy for both dose rates used (Supplementary Figure S3). Probably, the most radioresistant subpopulation in line #1 predominates compared to line #83. Moreover, various studies have shown that RT ionizing radiation is more effective in killing rapidly proliferating tumor cells than slowly dividing (i.e., quiescent or dormant) cells and that quiescence is associated with relative radiation resistance [42,43]. In our study, cell line #1 has a doubling time of 4.7 days compared with 2.2 days for line #83. As a result, less-proliferating #1 cells can survive irradiation generating a radiation-resistant fraction (i.e., quiescent population), which is appreciated at absorbed doses higher than 10 Gy.

Nevertheless, the patient associated with line #83 has a “very poor” prognosis concerning the patient associated with line #1, suggesting that radio resistance is not the only parameter associated with RT outcome. Other factors may promote tumor recurrences, such as a worse ability to repair DNA damage or a different genetic signature.

For this reason, we studied the DNA-damage induction and repair using the γ-H2AX assay. Our results demonstrate a different radiation-induced response between line #1 and line #83 (see also Figure S3). The former shows a high phosphorylation level, regardless of the dose rate used. In contrast, the latter shows a low level of histone γ-H2AX and differences in the slope of the dose–response curves, strongly dependent on the dose rate. It has been known for several years that the phosphorylation of histone H2AX on serine 139 by protein kinases of the phosphoinositide kinase family (including ATM, ATR, or DNA-PK) allows the recruitment of molecules involved in the signaling and repair of DNA breaks. To explain the differences observed in the γ-H2AX dose–response curve, we analyzed the expression of genes involved in cell signaling and DNA repair in the control cells, and we found that the expression of the HP1-b gene is downregulated in line #83 compared to line #1 (866.710.399 and 8.655.441.135 a.u., respectively).

It has been reported by Ayoub et al. [44] that following DNA damage, phosphorylation on the amino acid Thr51 of HP1-b, (a chromatin factor bound to histone H3), by CK2 (a protein involved in DNA-damage signaling) determines the mobilization of HP1-b itself from chromatin. These changes lead to a dynamic change of chromatin itself that, in turn, facilitates H2AX phosphorylation in mammalian cells. We can speculate that the downregulation of the HP1-b gene observed in line #83 affects the chromatin compactness making the molecules of histone H2AX surrounding a radiation-induced DNA DSB less accessible to the protein kinases of the phosphoinositide kinase family responsible for H2AX phosphorylation. The different chromatin structure is also corroborated by side scatter and forward scatter data from flow cytometry, which showed a different density of nuclei, more condensed in line #83 than in line #1 (Figure S4).

It remains to explain the big difference in the slopes of the dose–response relationships for DNA damage observed in line #83 as a function of the dose rate. A possible explanation could rely on the local chromatin relaxation induced by DSBs at the chromatin-damaged site. Biochemical evidence reported that chromatin structure is remodeled immediately after exposure to ionizing radiation due to radiation-induced DNA strand breaks that remove topological constraints on DNA loops [45]. Accordingly, it is reasonable to assume that at the same dose, the damage induced in a short window of time (high dose rate) can relax chromatin more efficiently than the same damage induced in a broader window of time (low dose rate). Consequently, the decondensation of chromatin establishes an accessible subnuclear environment that facilitates DNA damage signaling and repair [46,47].

Interestingly, spatio-temporal dynamics of chromatin restructuring were visualized during DNA damage response after high-LET and low-LET irradiation. The former is more effective in inducing clustered DNA damage, which triggers profound changes in chromatin structure along particle tracks. The latter mostly induces single DNA lesions throughout the cell nucleus, which do not lead to visible chromatin decompaction [48]. Similarly to spatially clustered damage along high-LET radiation tracks, temporally concentrated (high dose rate) damage may also be thought to cause rearrangements in chromatin architecture, which may affect its structural and functional organization.

This dose rate-dependent effect could not significantly affect the response of #1, possibly due to other mechanisms related to the presence of genes involved in chromatin dynamics (e.g., HP1-b).

It has also been suggested that chromatin relaxation and ATM activation reinforce each other, forming a positive feedback loop [48]. KAP-1 promotes chromatin condensation while pKAP-1 promotes chromatin decondensation (relaxation). Therefore, pKAP-1 enhances the local chromatin relaxation induced by DSBs at the chromatin damage site, and pKAP-1 spreads throughout the nucleus. Additional investigations are necessary to identify agents that induce differential amounts of DNA damage and ROS.

The positive and negative enrichment pathways of line #83 versus line #1 are attributable to their different metabolic fingerprint [40] and likely explain the differences in different baseline the total fluorescent intensity at baseline and the induction after the dose delivery with different dose rates using FFF beams.

Our findings were compared with those derived from public databases derived from GBM patients in terms of overall survival by grouping patients with gene signatures identified by top- and bottom-enriched pathways in the investigated GSC lines. These findings, potentially supporting a treatment strategy based on gene signature for stratifying GBM patients who could have an advantage from FFF-based treatment, should be confirmed in a prospective pilot clinical trial.

## 4. Materials and Methods

### 4.1. Cell Lines

V79 Chinese hamster lung fibroblasts and two primary human GSCs, named line #1 and line #83, from the number assigned to the biopsy from which they were isolated, have been used in this study.

V79 cells were grown in ‘Eagle’s MEM medium supplemented with 10% fetal calf serum, 2 mM glutamine, 50 U/mL penicillin, and 50 mg/mL streptomycin and maintained in a humidified incubator at 37 °C and 5% CO_2_. The cells had a plating efficiency (PE, i.e., the percentage of cells initially seeded into a flask that has formed a colony) of ~90% and a doubling time of about 12 h.

GSCs line #1 and line #83 were obtained through mechanical dissociation of surgical specimens from patients subjected to craniotomy at the Institute of Neurosurgery, Catholic University School of Medicine, Rome. Informed consent was obtained from the patients before surgery. All patients provided written informed consent according to the research proposals approved by the Ethical Committee of the Catholic University School of Medicine, UCSC (Prot. 4720/17). The patients showed similar tumor location, gender, and age but had different clinical outcomes. Line #1 derives from a patient with 6-month progression-free survival (PFS) and 12.5 months OS, representative of a “poor” outcome; line #83 derives from a patient with 3 months PFS and 6-month OS, representative of a “very poor” outcome. Both cell lines were established at the Department of Oncology and Molecular Medicine, Istituto Superiore di Sanità, Rome, and maintained in a serum-free medium supplemented with growth factors (EGF and b-FGF) as previously described [49].

The stemness of the GSC lines was validated by self-renewal capacity and expression of stem-cell markers, such as CD133, Sox2, Musashi, and nestin, other than by differentiation capacity assayed by co-expression, under serum stimulation, of astrocytic and neuronal phenotypic markers in vitro. The in vivo tumorigenic potential of GSCs was assayed by intracranial cell injection into immunocompromised mice, resulting in tumors with the same antigen expression and histological tissue organization as the human parent tumor [49,50].

The two GSC lines show differences in their growth characteristics. Line #1 cells, representative of the pro-neural-like molecular subtype [40], grow in suspension and have a doubling time of nearly 4 days. Line #83 cells, representative of the mesenchymal-like molecular subtype [40], present two components in balance with each other—one growing in suspension, the other one adherent to the flask surface—and they have a doubling time, evaluated on the overall culture, of about 2 days.

### 4.2. Irradiation Geometry, Treatment Planning, and Dose Measurements

In the present study, we have used a 10 MV TrueBeam^TM^ linear accelerator located at the Regina Elena National Cancer Institute (Rome) to investigate the radiobiological effect of different dose rates and pulse repetition rates on cellular responses.

FF and FFF beams were delivered at 4 Gy/min, while irradiations at 24 Gy/min were performed only with the FFF beam.

**Figure 6:**
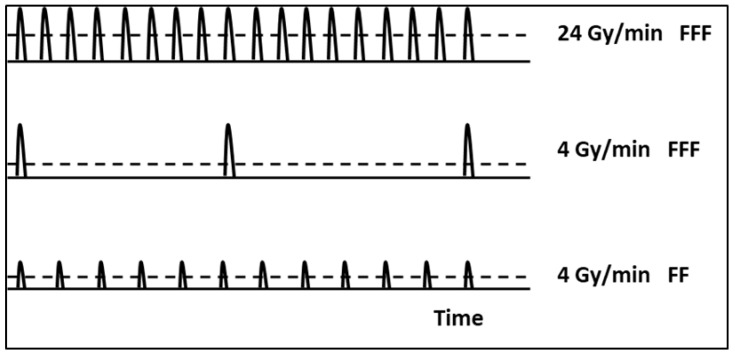
Schematic representation of pulse rates giving the same mean dose in different beam configurations (10 MV TrueBeam).

Beam characteristics are reported in Table 5 and Figure 6.

In summary, the two beams differ in their radiometric characteristics: at the dose rate of 4 Gy/min FF and FFF differ in both dose per pulse (DPP) and pulse repetition frequency (PRF), while the FFF beams at the dose rates of 4 or 24 Gy/min differ in PRF only.

**Figure 7:**
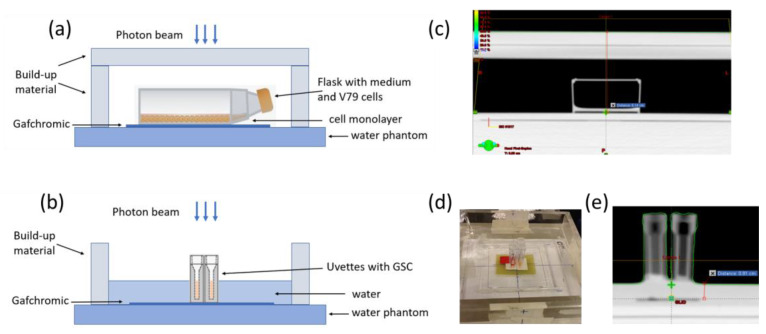
Experimental setup for V79 (**a**,**c**) and GCS cells (**b**,**d**,**e**).

Considering that the beam modulation performed by Verbakel et al. [10] to homogenize the dose on a large field of 10 × 10 cm^2^ reduces the effective dose rate, our setup was based on fixed fields with a maximum size of 4 × 4 cm^2^, as reported in Figure 7. The main effect of removing the FF in the 4 × 4 cm^2^ field is an overall softening of the energy spectrum over the irradiated area and a fourfold increase in the photon fluence.

V79 cells were irradiated as monolayers in tissue culture flasks (T-12.5 cm^2^). Irradiation of samples was performed at a source-to-cell distance of 100 cm. A 2 cm slab of RW3 plates were placed on top of the culture flasks to provide the build-up for every beam in the V79 setup (SSD = 94 cm), as shown in Figure 7a,c. Irradiation was carried out at room temperature (r.t.).

GSCs were centrifuged and then irradiated as pellets using 1 mL polystyrene cuvettes (Uvette, Deltalab) as commercial sample holders (Supplementary Material, Figure S5). Through centrifugation, the cells were sedimented at the funnel-shaped bottom of the cuvettes having the dimension 2 mm × 10 mm × 4 mm, l*w*h. We used the same setup as in previous works [51,52] on the radiobiological response of the same GSCs lines irradiated with differently charged particles (i.e., protons and C-ions) in the dose range of 5–40 Gy. This choice allows us to compare the results obtained after irradiation with different radiation qualities at different facilities.

In the present irradiation conditions, the cell culture medium and the plastic material forming the top of the vials used to irradiate GSCs were considered sufficient to guarantee the build-up for the setup (SSD = 99 cm) as shown in Figure 7b,d,e.

Both flasks with V79 cells or Uvette with GSCs were placed in a square container filled with about 2 cm water on top of 10 cm of water equivalent RW3 plates (PTW, Freiburg, Germany) to ensure full scatter conditions. This experimental setup allows for reducing the effect of lateral disequilibrium.

This beam setup guarantees homogenous irradiation of our cell samples, as demonstrated using the treatment planning system (TPS) and gafchromic films (see the next paragraph).

### 4.3. Dose Measurements—Dosimetry

Dose measurements were performed using gafchromic MD-V3 film, which had been tested in a wide dose range, namely 1–100 Gy [53,54]. Using the Truebeam accelerator (Varian Medical Systems, Inc., of Palo Alto, CA, USA), we constructed a calibration curve by irradiating films with known doses of 0, 1, 3, 5, 10, 15, 20, 30, 40, and 50 Gy. The linearity of dose and exposure with Monitor Units (MU) were also checked.

CT scans of every setup configuration were acquired with a LightSpeed™ Pro16 (General Electric Medical Systems, Waukesha, WI, USA) and fed to a TPS (Eclipse, Varian Medical Systems, Inc., of Palo Alto, CA, USA) to calculate the MU required for the irradiation of each sample. Gafchromic films were placed under the holder samples (flask or uvette) to check the accuracy of the dose delivery.

### 4.4. Cell Killing and Genomic Instability in V79 Cells

Early and delayed reproductive cell death was measured in V79 cells with the clonogenic survival assay according to the standard protocol for cells growing as monolayers.

“Early survival” refers to the classic clonogenic test [55], and “late survival” refers to the reduction in the clonogenic potential of the progeny of cells that survive radiation exposure, i.e., the occurrence of lethal mutations. This effect is likely induced by genomic instability in the cell population [19,20].

V79 cell monolayers were irradiated at two different dose rates: 4 Gy/min (with both FF and FFF beams) and 24 Gy/min (with FFF beam only), in the dose range of 1–10 Gy. Irradiations were performed on exponentially growing cells seeded 24 h before irradiation into 12.5 cm^2^ flasks at a density of about 8 × 10^3^ cells/cm^2^.

After irradiation, cells were trypsinized, counted (using a Coulter Counter Z2 serie Beckmann), diluted, and seeded into 25 cm^2^ flasks at the appropriate concentration to score a number of colonies of about 200 per flask for each dose. The flasks (5 for each dose point) were then incubated under standard culture conditions—37 °C in a 5% CO_2_ atmosphere—for 7 days. To evaluate the early reproductive cell death, colonies from 4 flasks were stained and counted to calculate the PE.

To evaluate the delayed reproductive cell death, the fifth flask was trypsinized and the cells were harvested, counted, and plated at the clonal density of 200 cells per T25 flask; 5 flasks per dose. The flasks were incubated under standard culture conditions for 7 days of growth before staining and scoring the colonies to calculate the PE for delayed reproductive cell death.

The experimental PE measured at the various doses D (including D = 0 Gy) were fitted with the function PE (D) = PE(0)_extr_ exp (−mD−nD^2^) for the early reproductive cell death (linear quadratic response), and with the function PE (D) = PE(0)_extr_ exp (−mD) for the delayed reproductive cell death (linear response), where PE(0)_extr_, m and n are free parameters. Then, for each experiment, cell surviving fractions, S(D), were calculated as the ratio between the values of PE(D) and PE(0)_extr_ obtained from the fit.

In the case of early reproductive cell death, the mean surviving fractions S(D) from independent experiments were fitted with the following function:

S(D) = exp (−αD−βD^2^) (1)

In the case of delayed reproductive cell death, the function used was:

S(D) = exp (−αD)(2)

### 4.5. Cell Killing in GSCs

GSCs cells were seeded in T175 cm^2^ flasks at a density of about 2 × 10^4^/mL for #83 and 4 × 10^4^/mL for #1, 7 days before irradiation in the dose range 5–40 Gy with two different dose rates, 4 or 24 Gy/min, both delivered in the FFF regimen. Before irradiation, both cultures were trypsinized to obtain homogenous cell suspensions to be irradiated after centrifugation inside Uvettes, as previously described.

Cell killing following irradiation was measured using the limiting dilution assay [56]. Briefly, after irradiation, cultures were diluted at the appropriate concentration and seeded in 96-well flat-bottom plates, two plates per sample, using the Transtar system (TRANSTAR-96, COSTAR). Dilutions of cell suspensions were made to seed each of the 96 wells with one cell per well for unirradiated and 5 Gy irradiated samples, and three cells per well for 10, 20, and 40 Gy irradiated samples. The plates (2 for each dose point) were then incubated under the standard culture conditions of 37 °C in a 5% CO_2_ atmosphere.

A fresh medium was added to the wells once a week, and the scoring of survivors was performed by microscopy about a month after seeding. Wells positive for survival were defined as those containing either neurospheres, aggregates, or single-viable cells. PE was calculated as described in [56].

The surviving fraction S(D) was calculated as the ratio between the PE at dose D and the PE of unirradiated control cells.

GSC survival curves show a biphasic trend. In the hypothesis that this trend is due to the presence of one or more subpopulations with different radiation sensitivity (see Results), the curves were fitted using the following mathematical expression:

S(D) = f * exp(−α_s_D−β_s_D^2^) + (1−f) * exp(−α_r_D−_r_D^2^) (3)

where f is the fraction of the “sensitive” cells, α_s_ and β_s_ are the linear and quadratic parameters for the “sensitive” population, and α_r_ and β_r_ are the linear and quadratic parameters for the “resistant” one (e.g., [23,24,25]).

However, since the survival data are confined within two decades of cell killing, Equation (3) is over-parameterized. Therefore, we introduced the assumption that both sub-populations produced a linear exponential response (β_s_ = 0 and β_r_ = 0) and used the following equation:

S(D) = f * exp(−α_s_D) + (1−f) * exp(−α_r_D) (4)

### 4.6. Flow Cytometry Measurements for γ-H2AX Detection in GSCs

Quantification of cellular DNA damage (in terms of DSB) induction, and repair, was evaluated by flow cytometry γ-H2AX assay, according to a protocol modified by Hamasaki et al. [57].

The γ-H2AX assay is based on the phosphorylation of many molecules (up to 2000) of histone H2AX at Ser139 occurring after DSB induction in the cellular DNA [58]. Accordingly, DSBs can be detected by immunofluorescent techniques using specific antibodies directed against phosphorylated Ser139. Fluorescence intensity, measured by flow cytometry after 2 h from irradiation, correlates with the DSB yield.

The Persistence Ratio (PR), defined as the percentage of the fluorescence intensity at the time (t) compared to the maximum fluorescence intensity (obtained at 2 h and representing the initial damage), was calculated to quantitively compare the repair in the two lines at the two dose rates (4 Gy/min and 24 Gy/min).

Briefly, cells were irradiated at r.t. in a culture medium and then incubated at 37 °C for 2, 4, and 24 h. At the end of the various incubation times, 3 × 10^5^ GSCs from each sample were fixed in 70% ethanol at r.t. and kept at +4 °C until performing the assay.

Fixed cells were seeded in 96-wells U-bottom plate (NUNC), washed twice in DPBS, centrifuged at 450 g for 5 min, and resuspended in 100 μL Permeabilization Buffer (PB: 1% BSA (*w*/*v*) and 0.2% Triton X-100 (*v*/*v*) in DPBS). Then samples were incubated with 100 μL of mouse monoclonal anti-phosphohistone H2AX (Ser139) antibody (Upstate) diluted 1:1000 with PB for 30 min at r.t., washed with PB and then incubated for 20 min at r.t. in the dark with 80 μL of secondary antibody (Alexa 488 F(‘ab’)_2_ goat antimouse, Molecular Probes) diluted 1:100 with PB.

Phosphorylation levels were determined by flow cytometry in terms of the area of the fluorescence intensity distribution (total fluorescence intensity). Data were analyzed using FlowJo software version 8.8 (Tree Star).

### 4.7. Gene Expression Profiling in GSCs

For GSC gene expression data collection, total RNA was extracted, labeled, and hybridized to the Affymetrix Gene Chip1.0ST array (Affymetrix, Santa Clara, CA, USA) according to the ‘manufacturer’s instructions. Data pre-processing before the formal statistical analysis involved standard processes of normalization, named the Robust Multi-array Average (RMA) method.

Gene expression was used for a Gene Set Enrichment Analysis (GSEA 4.1.0 software) to get the positive and negative enrichment pathways of line #83 versus line #1. The analysis was conducted in a pre-ranked mode by using sorted FCs of each gene between the two lines.

#### 4.7.1. The Cancer Genome Atlas (TCGA)

A normalized gene expression of the GSCs was obtained from Broad Institute TCGA Genome Data Analysis Center (2016): TCGA data from Broad GDAC Firehose 28/01/2016 run. Broad Institute of MIT and Harvard. Dataset. https://doi.org/10.7908/C11G0KM9 (http://gdac.broadinstitute.org/runs/stddata__2016_01_28/data/OV/20160128/, accessed on 23 September 2021).

All genes included in the positive and negative enriched pathways from gene expression analysis between the two investigated GSCs used to identify a virtual cohort from the TCGA GBM database.

#### 4.7.2. Target Prediction and Gene Set Enrichment Analysis (GSEA)

A preranked GSEA (https://www.gsea-msigdb.org/gsea/index.jsp, accessed on 23 September 2021) was performed on a list of genes modulated between two cell lines showing different responsiveness to RT. The GSEA algorithm calculates an enrichment score reflecting the degree to which the genes included in a gene set are over-represented at the top or bottom of the ranked list of all genes included in the expression dataset. The ranking was obtained based on the FC of each gene. GSEA was run in pre-ranked mode using classic as metric and 1000 permutations selecting the curated gene sets of Molecular Signatures Database (MsigDB) derived from Hallmark, KEGG, Reactome, and Wikipath database. Gene sets enrichment was assessed by positive and negative normalized enrichment score (NES) and false discovery rate.

### 4.8. Statistical Analysis

Statistical differences between the results obtained from different irradiation modalities for the various endpoints (cell killing and DNA damage assay) were analyzed using the Student’s *t*-test. Overall survival was evaluated by the Kaplan–Meier method and a log-rank test was used to establish the statistical significance of the distance between curves.

The impact of clinical variables on the survival curves was investigated by Cox proportional hazard regression model. The curves were defined based on positive and negative z-scores of the signal intensity of each gene. Analysis was conducted using MATLAB R2020b (The MathWorks Inc., Natick, MA, USA).

## 5. Conclusions

Our study confirms the advantage of using high-dose-rate exposure in clinical practice: V79 cell data support the use of FFF not only based on the reduced exposure time that increases the comfort of the patient but also based on the reduced capability of inducing genomic instability. Furthermore, for the first time in literature, we showed several differences in GBM patient-derived stem-like cells, which allowed us to identify patients with candidate gene signatures that are expected potentially to benefit from FFF beam irradiation. Moreover, we linked the observed biological effects on the overall survival of GBM patients derived from the TCGA GBM database, grouping the virtual cohort using candidate signatures, thus identifying GBM patients that could benefit from RT treatments delivered with FFF beams. Further studies are recommended to elucidate this aspect in the clinical setting.

## Figures and Tables

**Table 1:** Parameters of the dose–response curves for the early and late survival of V79 cells irradiated with 10 MV photons in the different beam configurations.

		Early Survival		Late Survival
Beam	Dose Rate (Gy/min)	α (Gy^−1^)	β (Gy^−2^)	α (Gy^−2^)
10 MV FF	4	0.084 ± 0.037	0.025 ± 0.006	0.084 ± 0.015
10 MV FFF	4	0.124 ± 0.036	0.019 ± 0.005	0.080 ± 0.021
10 MV FFF	24	0.166 ± 0.035	0.017 ± 0.005	0.034 ± 0.013

**Table 2:** Radiobiological parameters of the dose–response curves for cell killing of GSCs irradiated with 10 MV photons in the different beam configurations.

Beam	Dose Rate (Gy/min)	Line	f	α_s_ (Gy^−1^)	α_r_ (Gy^−1^)
10 MV FFF	4	#1	0.56 ± 0.30	0.24 ± 0.29	0.04 ± 0.02
10 MV FFF	24	#1	0.80 ± 0.23	0.19 ± 0.13	0.00 ± 0.04
10 MV FFF	4	#83	0.88 ± 0.08	0.27 ± 0.11	0.02 ± 0.02
10 MV FFF	24	#83	0.93 ± 0.05	0.24 ± 0.05	0.00 ± 0.02

**Table 3:** PR of total fluorescence intensity after exposure to 20 Gy with FFF beam at different dose rates for GSC line #1 and line #83.

Beam	DR (Gy/min)	Line	PR (%) @ 4 h	PR (%) @ 24 h
10 MV FFF	4	#1	45 ± 17	4 ± 3
10 MV FFF	24	#1	45 ± 18	24 ± 14
10 MV FFF	4	#83	50 ± 27	6 ± 4
10 MV FFF	24	#83	64 ± 20	14 ± 2

**Table 4:** List of relevant publications related to the different cell lines, end-points, irradiation setups, and effects discussed in the main text.

Cells	End-Point	E (MV)	Dose Rate (Gy/min)	Dose (Gy)	Modulated Beam	Observed Effect	Reference
HNCa, FaDu_DD_; Chinese hamster lung fibroblasts, V79	Clonogenic cell survival	6 FFF	5.01, 9.99, 29.91	1–10	No	No	Sørensen et al. [9]
		6 FF					
GBM cell lines: T98G (mut-p53) and U87MG (wt-p53)	Clonogenic cell survival	10 FFF	4, 24	5, 10	No	Yes *	Lohse et al., 2011 [16]
		10 FF	0.2, 4, 6				
Cervical carcinoma SiHa; NSCLC H460; V79	Clonogenic cell Survival, γ-H2AX induction	6 FFF	3.6, 10	2, 5, 10	Yes (compensator)	No	Karan et al., 2013 [12]
		6 FF	3.6		No		
SCLC SW1573; GBM cell lines T98 (mut-p53); astrocytoma D348	Clonogenic cell survival	10 FFF	~24	2–12	Yes (IMRT)	No	Verbakel et al., 2013 [10]
		6 FF	~5.8				
GBM cell lines T98G (mut-p53); U87MG (wt-p53), V79	Clonogenic cell survival	10 FFF	4, 24	5, 10	No	No	Lasio et al., 2014 [14]
		6 FF	4				
		6 FFF	4, 14				
Human Ca lung A549; Ca breast (MCF and Ca brain U373 MG, Ca colon HCT116 and DLD-1; normal human lung NL20 and breast MCF10A; CHO9	Clonogenic cell survival	10 FFF	4, 24	2, 5, 10	No	No	Dubois et al., 2015 [33]
		10 FF					
GSCs	Several ^#^	6 FFF	0.2, 4, 4.2, 21.2	2–6	No	No	Hao et al., 2018 [34]
		6 FF					
Colon Ca CT26 murine	Several ^§^	10 FFF	4, 12, 24	2–12	No	No	Laurent et al., 2020 [35]
		10 FF					
Melanoma cell lines (WC00046, WC00060, and WC00081)	Clonogenic cell survival	10 FFF	4, 24	0.25–8	No	Yes	Sarojini et al. [17]

* at Doses ≥ 10 Gy; ^#^ cell viability, tumor sphere formation, clonogenic cell survival, cell cycle, apoptosis, and DNA damage. ^§^ in vitro: Anti-tumor immune response; in vivo: tumor growth retardation, composition of immune cell infiltrates within tumor microenvironment and the expression of immune checkpoints in immunomonitoring and RNAseq. Abbreviations: Ca: cancer, HNCa: head-and-neck cancer; PCa: prostate cancer; SCLC: small-cell lung cancer; NSCLC: non-small-cell lung cancer; GBM: glioblastoma; Chinese hamster ovary: CHO.

**Table 5:** Beam Characteristics.

Energy	Q-Index	DPP (cGy/Pulse)	Pulse Length (µs)	PRF (Hz)	Average Dose Rate (Gy/min)	Delivery Time for 5 Gy (s)
10 MV FF	0.735	0.028	4.5	240	4	75
10 MV FFF	0.691	0.111	4.5	360	24	12.5
			4.5	60	4	75

## Data Availability

The data presented in this study are available on request from the corresponding author. The data are not publicly available due to limitations of Italian law.

## Supplementary Materials

The following supporting information can be downloaded at: https://www.mdpi.com/article/10.3390/ijms24021107/s1. References [40,49,59] are cited in the supplementary materials file.

## Abbreviations

NCOA4	Nuclear Receptor Coactivator 4
NBN	Nibrin
STAG2	Stromal Antigen 2
MAPRE1	Microtubule Associated Protein RP/EB Family Member 1
E2F5	E2F Transcription Factor 5
PTEN	Phosphatase and tensin homolog
CEP57	centrosomal protein 57
GALNT12	Polypeptide N-Acetylgalactosaminyltransferase 12
POR	Cytochrome P450 Oxidoreductase
RPL39L	Ribosomal Protein L39 Like
CUL1	Cullin 1
MAP2K3	Mitogen-Activated Protein Kinase Kinase 3
CAMK4	Calcium/Calmodulin Dependent Protein Kinase IV
DENND2A	DENN Domain Containing 2D
HPS1	Hermansky-Pudlak syndrome 1
ADAMTSL1	a disintegrin and metalloproteinase with thrombospondin like protein 1
SCO1	Synthesis Of Cytochrome C Oxidase 1
GPC5	Glypican 5
GALNT6	Polypeptide N-Acetylgalactosaminyltransferase 6
MYO1C	Myosin IC
RT-qPCR	quantitative real time PCR
NMR	Nuclear Magnetic Resonance
HP1-b	heterochromatin protein 1-beta
CK2	casein kinase 2
LET	Linear Energy Transfer
KAP-1	Transcription intermediary factor 1-beta
ROS	Reactive oxygen species
EGF	Epidermal Growth Factor
b-FGF	basic Fibroblast Growth Factor
SSD	Source Surface Distance
CT	Computed Tomography
DPBS	Dulbecco’phosphate-buffered saline
BSA	Bovine Serum Albumina
KEGG	Kyoto Encyclopedia of Genes and Genomes
