# Supplementary material for: Effectiveness of Flattening-Filter-Free versus Flattened Beams in V79 and Glioblastoma Patient-Derived Stem-like Cells
Source: Int J Mol Sci. 2023 Jan 6;24(2):1107. doi: 10.3390/ijms24021107 (PMC9861147; doi:10.3390/ijms24021107)
Supplement: Supplementary file 1 [file ijms-24-01107-s001.zip › ijms-2043403-supplementary/Supplementary figures.pdf]

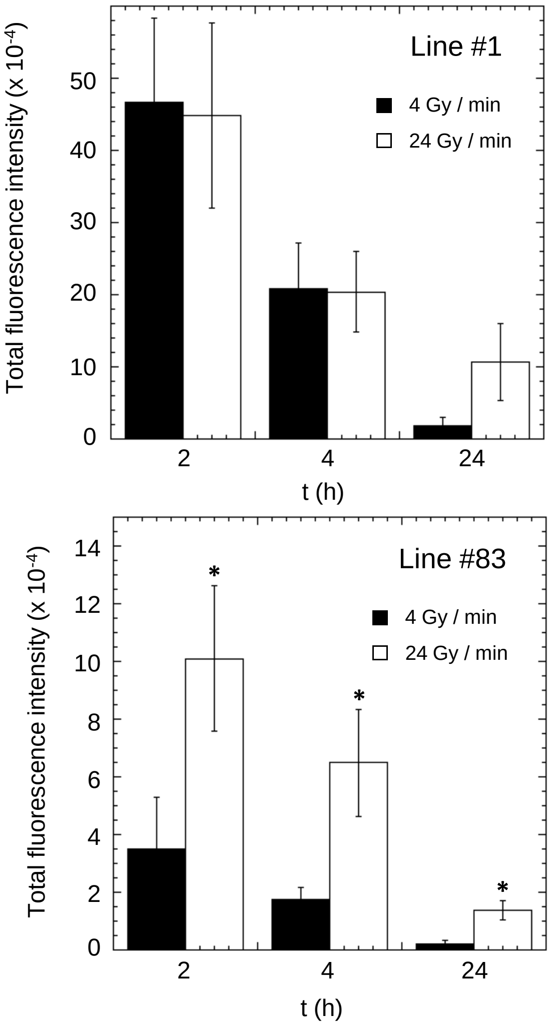

Figure S1. Dephosphorylation of γ-H2AX in line #1 and line #83 after the dose of 20 Gy at 2, 4 and 24 h from irradiation. * $p < 0.01$

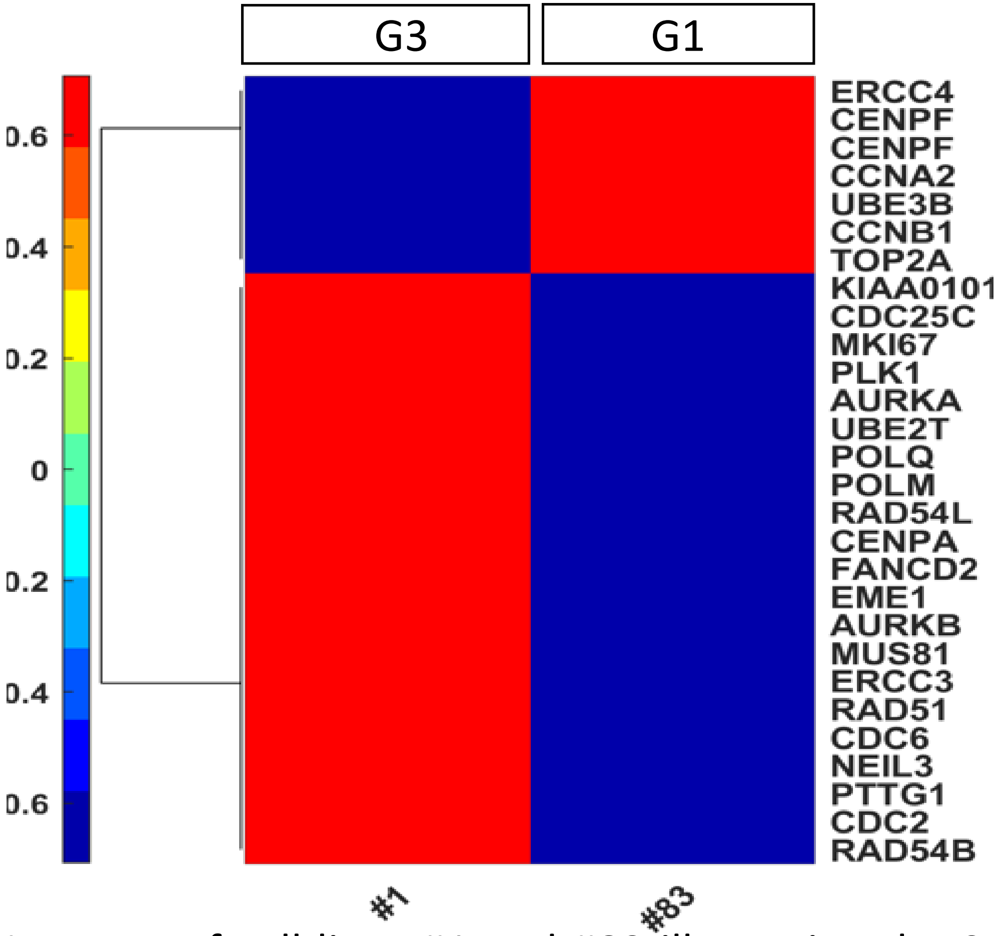

Figure S2. Heatmap of cell lines #1 and #83 illustrating the 2 components as defined by Gobin et al. Standardized expression values are depicted using a red (high) to green (low) color key.

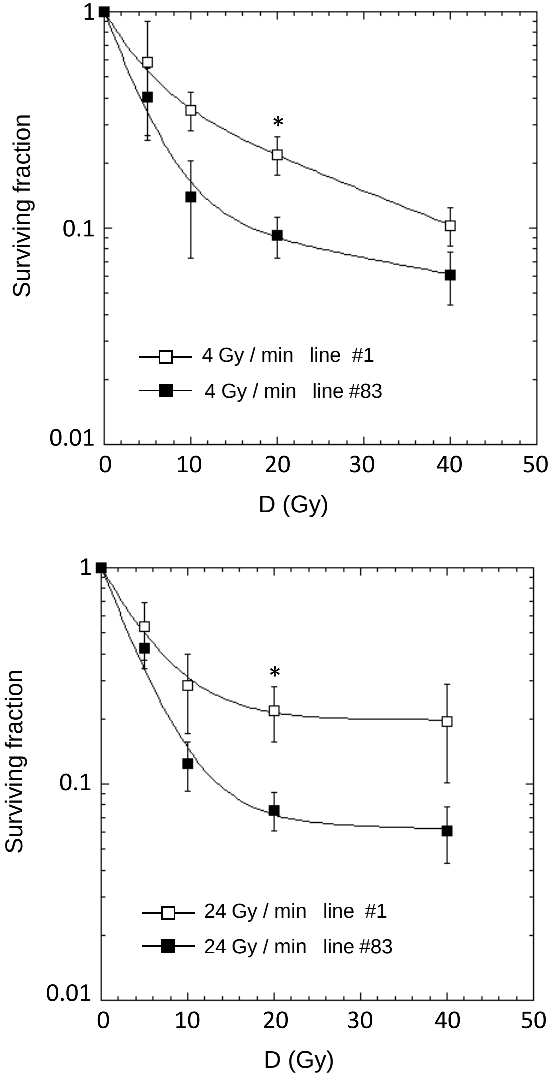

Figure S3: Dose-response curves for GSC cells irradiated in FFF conditions at different dose rates. The error bar represents the standard error of the mean (SEM) coming from at least 2 independent experiments for each irradiation condition used. * $p < 0.01$

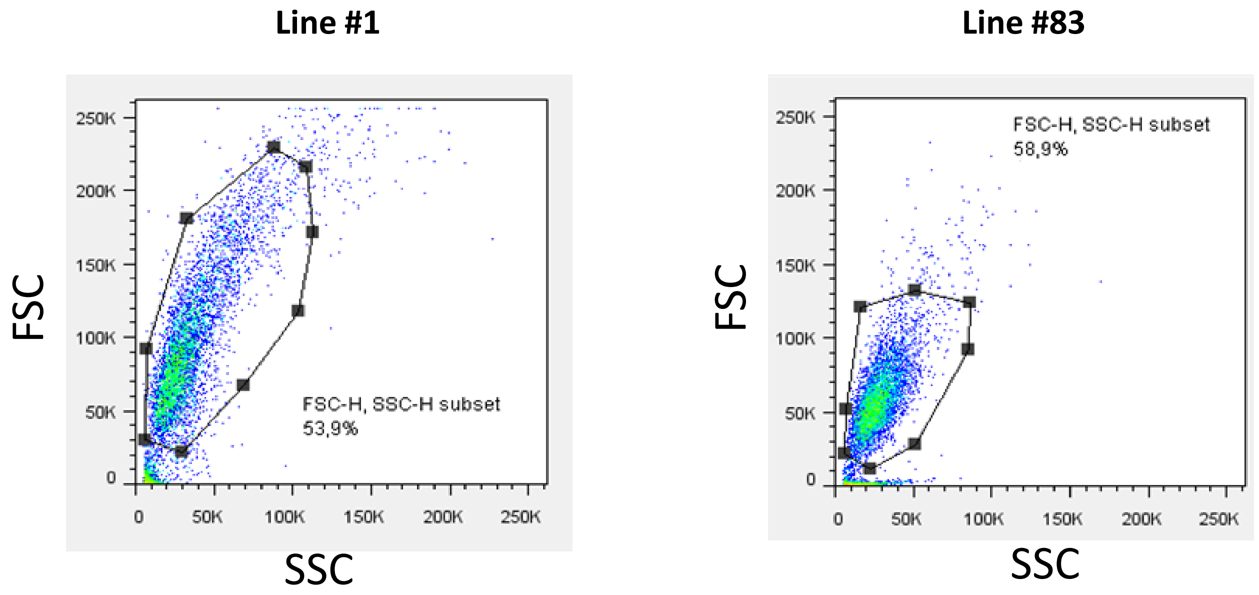

Figure S4. Flow Cytometry forward scatter (FSC) versus side scatter (SSC) of GSCs line #1 and line #83 cells. The former detects scatter along the path of the laser and indicates cell size. The latter detects scatter at a ninety-degree angle relative to the laser and provides information about a cell's internal complexity (i.e. granularity).

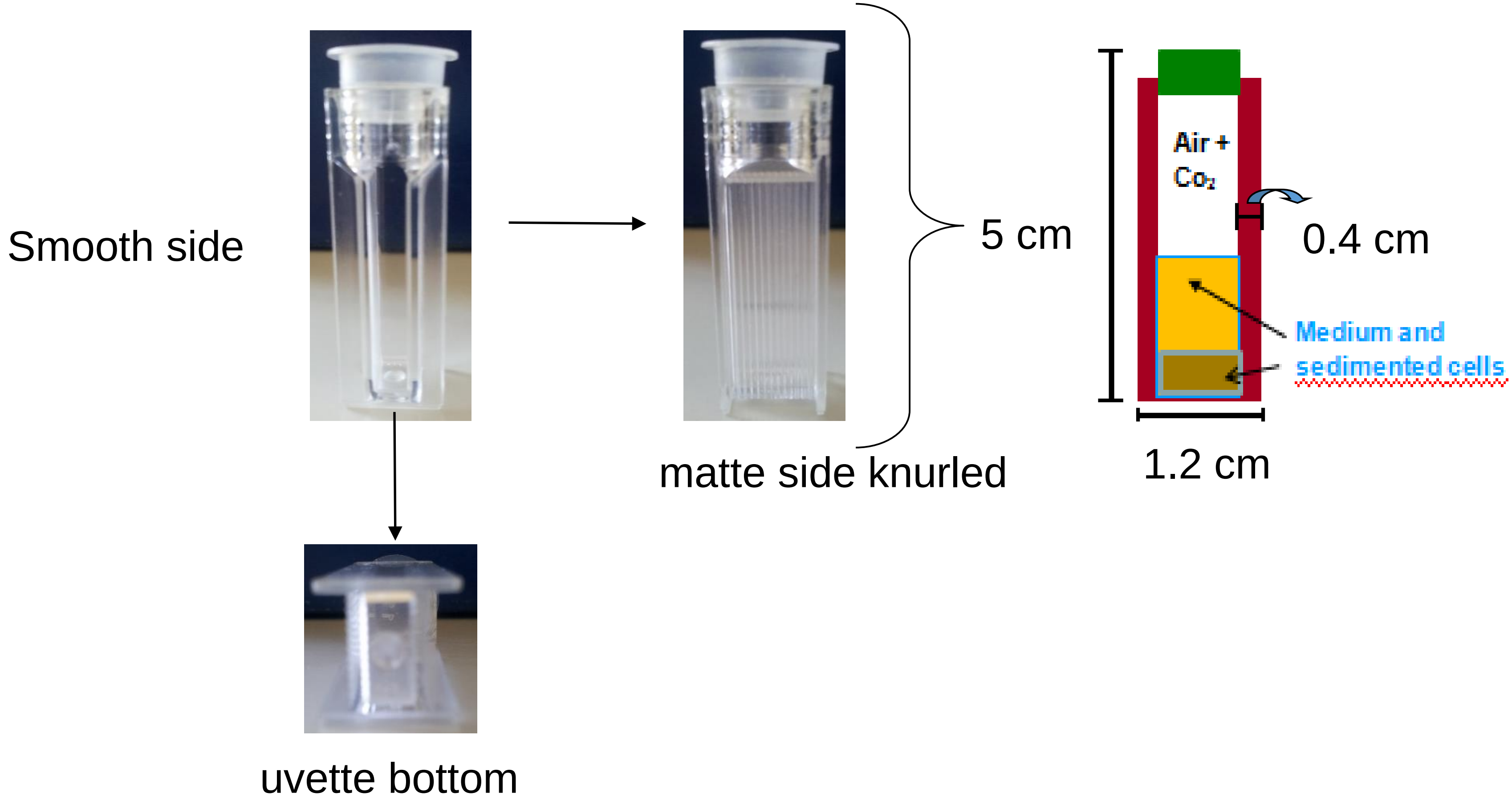

Figure S5. Uvettes used for irradiations at 10 MV TrueBeam™ linear accelerator located at the Regina Elena National Cancer Institute (Rome)
