# Supplementary material for: Effectiveness of Flattening-Filter-Free versus Flattened Beams in V79 and Glioblastoma Patient-Derived Stem-like Cells
Source: Int J Mol Sci. 2023 Jan 6;24(2):1107. doi: 10.3390/ijms24021107 (PMC9861147; doi:10.3390/ijms24021107)
Supplement: Supplementary file 1 [file ijms-24-01107-s001.zip › ijms-2043403-supplementary/Table S1.pdf]

Table S1. Patient characteristics and GSCs tumor biomarkers for lines #1 and #83.

	**#1**	**#83**	**Ref.**
Patient age (y)/sex (M/F)	40/M	52/M	[Pallini R et al. 2008]
Tumour location	Temporal close to SVZ	Temporal close to SVZ	[Guidoni L et al. 2014]
KPS (%)	80	70	[Pallini R et al. 2008]
CD133 (%)	>80	<1	[Guidoni L et al. 2014]
Ki67 (%)	20	40	[Pallini R et al. 2008]
PFS (m)	6	3	[Pallini R et al. 2008]
OS (m)	12.5	8.5	[Pallini R et al 2008]
Clonogenic index	1/4.32	1/2.66	[Guidoni L et al. 2014]
Sox2	+	+	[Guidoni L et al. 2014]
GFAP	+	+	[Guidoni L et al. 2014]
Tubulina Beta3	+	+	[Guidoni L et al. 2014]
Metabolic phenotype	Neuronal	Astrocyte-glioma	[Guidoni L. et al. 2014]
Gene expression/phenotype	proneural-like signature/GSf	mesenchymal-like signature/GSr	[Marziali G. et al 2017]
Growth pattern *in vitro*	floating spheres	adherent growth and small spheres	[Guidoni L. et al 2014]
Doubling time (d)	4.7	2.2	[Guidoni L. et al 2014]
p53 (by NGS)	Wild Type	Mutant in exon 5	[personal comunication]

KPS, Karnofsky performance status; PFS, progression-free survival; OS, overall survival; GFAP, Glial fibrillary acidic protein; GSf, glioblastoma full stem; GSr, glioblastoma restricted stem; NGS, Next Generation Sequencing

Pallini, R.; Ricci-Vitiani, L.; Banna, G.L.; Signore, M.; Lombardi, D.; Todaro, M.; Stassi, G.; Martini, M.; Maira, G.; Larocca, L.M.; De Maria, R. Cancer stem cell analysis and clinical outcome in patients with glioblastoma multiforme. *Clin. Cancer. Res*. **2008**, *14* (24), 8205-12. doi: 10.1158/1078-0432.CCR-08-0644.

Guidoni, L.; Ricci-Vitiani, L.; Rosi, A.; Palma, A.; Grande, S.; Luciani,, A.M..; Pelacchi F.; di Martino, S.; Colosimo, C.; Biffoni, M.; De Maria, R.; Pallini, R.;, Viti. V. 1H NMR detects different metabolic profiles in glioblastoma stem-like cells. *NMR Biomed*. **2014**, *27*(2), 129-45. doi: 10.1002/nbm.3044.

Marziali, G.; Signore, M.; Buccarelli, M.; Grande, S.; Palma, A.; Biffoni, M.; Rosi, A.; D'Alessandris, Q.G.; Martini, M.; Larocca, L.M.; De Marias, R.; Pallini, R.; Ricci-Vitiani, L. Metabolic/Proteomic Signature Defines Two Glioblastoma Subtypes With Different Clinical Outcome. *Sci Rep* **2016**, *6*, 21557. doi: 10.1038/srep21557.
